# Institutional Entrepreneurship in Loosely Coupled Systems: The Subject Position of MOOC Entrepreneurs and Their Interpretive Struggles in a Norwegian Context

**DOI:** 10.1007/s10758-023-09647-9

**Published:** 2023-05-09

**Authors:** Inger Dagrun Langseth, Dan Yngve Jacobsen, Halvdan Haugsbakken

**Affiliations:** 1 Department of Teacher Education, Norwegian University of Science and Technology, Trondheim, Norway; 2 Department of Education and Lifelong Learning, Norwegian University of Science and Technology, Trondheim, Norway; 3 Department of Education, ICT and Learning, Østfold University College, Halden, Norway

**Keywords:** MOOC, Micro-credentials, Support units, Higher education, Digital projects, Entrepreneurs, Pockets of innovation, Subject position

## Abstract

While technological change in organizations is fast and eminent to most people, the adoption of Massive Open Online Courses, micro-credentials, and flexible and scalable online courses, appear to be comparatively slow in Higher Education in the Nordic countries. To explore this phenomenon, we completed 10 qualitative interviews at ten different higher education institutions across Norway in fall 2020. The informants were strategically selected among employees who had been involved in open platform technology, MOOC production and support for faculties. Adopting thematic analyses, we found entrepreneurs who positioned themselves in pockets of innovation with the intention to transform teaching and learning. Rather than seeing technological innovations as “more of the same”, the entrepreneurs embraced the possibilities emerging in new educational practices. Inspired by *New Institutionalism*, we focused on the organizational conditions for MOOC production. The entrepreneurs often entered interpretive struggles at higher organizational levels in competition with other stakeholders. Despite national initiatives and funding, many stakeholders questioned the value of MOOCs. Our study points to discrepancies in understanding the disruptive and transformative change that new technology can bring to study programs and lifelong learning. The informants also experienced insufficient support from leaders and lamented the lack of a national platform for open online access. We link these findings to embedded theories, belief systems and discourses in educational cultures and management in Higher Education.

## Introduction

Rapid changes in society and demands for skills and aptitudes in professional life call for new pathways in education and lifelong learning. One way of responding to this demand is to offer flexible, scalable, stackable, and modular learning opportunities for learners online. In Higher Education Institutions (HEI), access to Massive Open Online courses (MOOC), study programs and micro-credentials (short courses with badges or ECTS), have grown considerably over the last decade (Class Central, 2021). This development is facilitated by new technology, such as open platforms, automated feedback, social and participatory media and video tools, which makes it possible for faculties to develop a social learning environment for a wide range of students online (Conole, 2012; Pei & Wu, 2019). The advances are mainly driven by early adopters and enthusiasts (Langseth et al., 2022; Tungesvik, 2021). The strategic move towards Open Educational Resources (OER) has also contributed to the possibility that faculty members can share and reuse courses and content they produce (Colvard et al., 2018; Mishra et al., 2022). According to Class Central (2022; 2023), online learning is stronger than ever, with hundreds of thousands of free courses worldwide. In this study, we explore the adoption of such online learning opportunities in HEIs, while focusing on MOOC entrepreneurial activities among staff and faculties in Norwegian HEIs.

MOOCs are different from long-established online course offerings on Learning Management Systems (LMS) and can generally be defined as *scalable online learning* on open platforms, involving network connectivity, artificial intelligence and machine learning (Moe, 2015). The open and inclusive mindset residing in MOOCs is not without challenges and controversy. On the one hand, there are many opportunities. MOOCs are democratic and contribute to equity in society (Pilli et al., 2018). Online tools can be used for collaborative learning, group work, projects, problem-solving and creative thinking to develop necessary aptitudes in the knowledge economy (Bates, 2010). Micro-credentials based on MOOCS can offer an added certification to an existing degree (Selvaratnam & Sankey, 2021), or they can be *stackable* into a larger course or study program (Ahmat et al., 2021). The *scalability* of MOOCs and micro-credentials makes it possible for learners to register and get access to the content and receive a certificate or some form of formal accreditation after completing a course (Kaplan & Haenlein, 2016). Learners have *flexibility* in their studies, meaning that they can study at their own pace, from wherever they are located, receive (automated) feedback, and collaborate with peers online. Learners, who are just interested in dropping in out of personal interest, can access course content, benefit from participating in discussion forums and reach their personal goals (Jacobsen, 2019). The *modulization* of online course content (OER) makes it possible to integrate MOOCs and micro-credentials in campus programs, or they may function as supplemental learning for students, who want an alternative perspective on their learning material (Rivard, 2013). The European Commission European Universities Initiative emphasizes the need for both virtual and on-campus learning in the development of new pathways for students in European HEIs (Arnaldo Valdés & Gómez Comendador, 2022). Learners from different geographical locations and backgrounds can document their competence, participate in academic educational discourses and develop their capabilities (Biesta, 2011). This move also reflects one of the sustainable development goals issued by the United Nations (cf. goal 4. Quality Education). Online learning cuts students’ expenses and involves, for example, considerably less energy and CO2 emissions than full-time campus-based courses (Roy et al., 2008). MOOC design also penetrates education on campus, where *blended learning* is recommended (JISC, 2020).

On the other hand, there are challenges related to the many aspects of the MOOC phenomenon. In a study examining the strengths and weaknesses of MOOCs, dropout rates, poor pedagogy, low quality assessment and little knowledge about students’ needs in online learning are generally considered barriers to their effectiveness (Pilli et al., 2018). When micro-credentials are integrated into study programs, they pose challenges related to *stackability*, meaning transparency in a holistic approach to learning outcomes and assessment (Boud & Jorre de St Jorre, 2021). Micro-credentials have also been criticized for adapting to personal motivation and *employability*, with a narrow focus on preparation for work. Hence, they reshape the classification and framing in HE curricula (Wheelahan & Moodie, 2021). Against this background, the development of strategies for education and support systems for faculty is an issue (McGreal & Olcott, 2022), also in Norwegian HEIs (Fossland et al., 2020; Langseth et al., 2021; Laterza et al., 2020; Stensaker, 2018; Tungesvik, 2021).

The COVID-19 pandemic has accelerated the already existing trend in online learning in HEIs (Boud & Jorre de St Jorre, 2021). A massive body of research on the effect of the COVID-19 pandemic in the 2020s underlines the role that new technology and digital skills play in paving the way for new opportunities for teaching and learning online (Carrillo & Flores, 2020; Pokhrel & Chhetri, 2021; Amhag et al., 2019; García-Morales et al., 2021; Skulmowski & Rey, 2020; Watermeyer et al., 2021; Zawacki-Richter, 2021). Online education faced, for example, challenges related to poor technological infrastructure and digital skills among students and faculty (Onyema et al., 2020). Support for the implementation of technology-based and pedagogically informed teaching and learning was also in high demand (Müller et al., 2021).

We find it useful to distinguish between the MOOC initiative on open platforms, which dates back to the first article on Connectivism in 2008 and the year of the MOOC in 2012 (Downes, 2020), and the remote emergency teaching that happened mainly on existing Learning Management Systems (LMS) and video conferencing tools during the pandemic in the 2020s. The impact that the explorative use of new technology in digital teaching and learning had on faculties and organizational contexts during the pandemic in the Nordic countries, supported the already ongoing transition to online education (Laterza et al., 2020). Remote teaching and learning during the pandemic are, however, outside the scope of this article. Likewise, the study of the effects of teaching, assessment and student active learning in MOOCs and micro-credentials have been examined elsewhere (Kizilcec et al., 2020; Pilli et al., 2018). In this study, we build on previous studies, where we explored the limitations related to the adoption of MOOCs and the role of support units in digital transformation in HEIs in Norway (Langseth et al., 2021; 2022). Here, we explore the subject position of MOOC entrepreneurs and their interpretive struggles, as they use their room for maneuver, form pockets of innovation and seek changes in teaching and learning in a Norwegian context.

## Theoretical Perspectives

To explain and position our study more accurately, a research perspective must be established. A well-defined research lens will enable us to pinpoint in what ways our study contributes to new knowledge or if it substantiates insights already known to educational researchers. Moreover, it helps us to justify the selected theoretical key concepts and relevant research streams in which our research engages. In so doing, we need a point of departure, meaning to account for updated and general research on MOOCs.

This positioning can be achieved by extrapolating important and overarching key themes from recent systematic research reviews of MOOC research. These research reviews synthesize published studies from the previous decade (2010–2020), and thus provide a great scope of the research field. Still, we find that drop-out, retention, and attrition issues spark great research interest (Chen et al., 2022; Chiappe & Castillo, 2021; Estrada-Molina & Fuentes-Cancell, 2022; Wang et al., 2022). These research reviews are prone to emphasizing different explanations as to why learners drop out of MOOCs, which can be attributed to ineffective online course design, lack of belonging, time factors, and hidden costs, etc. Even so, we observe that educational researchers are deeply engaged in exploring the pedagogical effectiveness of MOOCs from different perspectives. For example, research reviews examine conditions for self-regulated learning strategies, (Ceron et al., 2021), to what extent course creators use learner feedback to improve and change course design and learning contents and learning activities (Dalipi et al., 2021, Moore & Blackmon, 2022); whether there is a need for improving and scaling of peer-assessment in MOOCs (Dalipi et al., 2021), if MOOCs contribute to student equity and social inclusion (Lambert, 2020) and, finally, if discussion forums have the desired effects on social learning (Almatrafi & Johri, 2019). Among new research trends, we can observe that a new type of MOOC course format is emerging, the so-called language MOOCs (LMOOCs) (Díez-Arcón & Martín-Monje, 2022; Palacios Hidalgo et al., 2020). Beyond that, researchers make comprehensive overviews describing the conditions and development for MOOCs on national levels which among other have been completed for China (Cheng et al., 2022) and Malaysia (Albelbisi & Yusop, 2020).

On that note, our assessment of current MOOC research is that there are knowledge gaps. To our knowledge, there is not an established research stream that examines the organizational conditions for MOOCs at universities, moreover, which exists to theorize and connect MOOC research to digitalization and organizational theory and to frame MOOC research from a national perspective. Therefore, to formulate our theoretical perspective, we use concepts and perspectives from *New Institutionalism* in organizational research (DiMaggio & Powell, 1983; Meyer & Rowan, 1977), and connect them to emerging research on digitalization of the Norwegian HEI and MOOC in Norway. These perspectives are employed to frame entrepreneurial and organizational conditions for facilitating flexible and decentralized online education.

### Recent Research on Digitalization and MOOCs in Norway

Over the recent years, a research stream explores the effects on digitalization of education in HEI in Norway. The research points out that digitalization is somewhat under the influence of administrative and IT staff and includes, only to a very low degree, educators (Tømte et al., 2019). This argument has been further theorized by Bygstad et al. (2022), who suggest that digitalization is prevented by forces that impede development. On the one hand, Bygstad et al. argue that centralized stakeholders advocate one form of digitalization. Here, digitalization is primarily promoted by autonomous IT departments that implement and administer “off-the-self” learning technologies such as Learning Management Systems (LMS). These are actors, who are not always in conjecture with the target group they are set to serve, in this case the faculties. On the other hand, another form of digitalization can be found among faculties, who explore how digital technologies support pedagogical practices across disciplines. This understanding of digitalization is more concerned with the transition from campus to online pedagogies in the practice field. The major challenge is that the two perspectives are poorly integrated, leading to multiple forces that orbit with different understandings. In other words, we sense patterns of loose couplings (Weick, 1976) at play in educational systems where different actors belong to various organizational structures and operate under different logics and understandings.

Research om MOOCs in Norway has two distinct approaches that frame the focus of our research. The first approach relates to research on MOOCs in a policy, adoption, and diffusion perspective. Early government reports attempted to define what a MOOC ‘is’ or they distilled experiences from government funded projects that explored the MOOC concept at local universities (Koch, 2017; NOU, 2014). Furthermore, researchers also asked whether there is a separate Scandinavian model for MOOCs and whether government agencies had played a role in its success (Tømte et al., 2020). Hence, researchers constructed frameworks that explain how MOOCs are adopted and diffused. In Norway, the adoption and diffusion patterns are somewhat different from global approaches. Online courses are still primarily organized and hosted on proprietary LMS platforms at the local HEIs (Tømte et al., 2014, 2017, 2020), while few faculties have access to global MOOC platforms. The adoption and diffusion pattern for the ‘localization’ of MOOC initiatives is therefore distinctive for Norway. In the second approach, we identify research on how faculties are inspired by MOOC pedagogies and make their own variants that are offered to students in continuing and further education. Such online courses are often asynchronous, flexible, and scalable. An example of a framework that is often associated with MOOC inspired pedagogies is Teachers’ Professional Digital Competence (Lund et al., 2014). Here, researchers attempt to ascertain the pedagogical effectiveness of MOOC-inspired university courses (e.g.: Brevik et al., 2019; Engeness & Nohr, 2020; Engeness et al., 2020; Haugsbakken, 2020; Jacobsen, 2019; Langseth et al., 2018). A similar division, yet without mentioning the MOOC movement, is made by Bygstad et al. (2022) who focus on two streams of digitalization in higher education, the digitalization of education itself and the digitalization of subjects. They label this *dual digitalization* and claim that this has been an obstacle for digital transformation in the sector.

There are also shortcomings with the aforementioned studies. Researchers have so far not described the complex organizational conditions for realizing MOOC initiatives. In fact, faculty members with a passion for fully online education, that we have labeled *MOOC entrepreneurs*, face considerable challenges. They are often engaged in interpretive struggles with other stakeholders or powerful actors with institutional agency. These factors are barely addressed in the research (Haugsbakken & Langseth, 2017; Krokan, 2017). Already in 2017, Haugsbakken and Langseth (2017) argued, for example, that there was a need to outline a separate strategy for MOOCs at universities, and to align the organization for MOOC production and support systems for faculty, which involves a technological infrastructure and the alignment with legal requirements. Consequently, we can relate to the observations made by Bygstad et al. (2022) that there are multiple and unintegrated understandings of MOOCs. Stakeholders might, for example, have other or limited understandings, such as not knowing what a MOOC ‘is’, or they might use other labels, such as “flexible education and decentralized education” for up- and reskilling, implying understandings that are not in alignment with those held by MOOC entrepreneurs. The emergent research literature on MOOCs does not address organizational issues and conditions. This represents a gap where we aim to contribute with our research.

Furthermore, we wish to point to organizational processes that may impede or propel the development of digitalization in HE. In a previous study, we found that formalized and research-based support for MOOC production is still scarcely developed in Norway (Langseth et al., 2022). In this study, we pursue these perspectives further and go on to investigate organizational conditions for MOOC entrepreneurship.

### Subject Positioning and Interpretive Struggles

This overview of the research on MOOCs makes it more apparent why it is relevant to apply New Institutionalism. Although strategic discourses on MOOCs are ongoing, we argue that educational research still has grounds to cover in the investigation of how MOOC entrepreneurs organize themselves in and across universities. This aspect applies to the position they might occupy in a *social field* and how they attempt to initiate changes to transform education. In short, Bourdieu’s (1984, 1986) *social fields* are arenas where we find an ongoing production, circulation, and exchange of resources and where different actors occupy positions in their struggle and competition over different forms of capital. In this context, New Institutional theory offers a range of perspectives to understand how change agents, i.e., MOOC entrepreneurs, work. In this study, Institutional Entrepreneurship theory explains the agency of MOOC entrepreneurs (DiMaggio, 1988).

The rationale for using institutional entrepreneurship relates to early observations made by new institutionalists. Meyer and Rowan (1977) argued that organizations adopt rational and technical procedures to gain legitimacy among other organizations, implying that procedures and instruments intended to improve organizations turn into *rational myths*. Rational myths are generally defined as stories, language, symbolic actions etc. that serve to create meaning, inspiration, and credibility to instigate change in the organization. Leaders often use such myths to convince employees to initiate change processes and reach organizational goals (Irgens, 2016). Here, organizational discourses are superficial and serve little purpose to improve organizational life. In the process, the championship for legitimacy over other organizations leads to decoupling, giving organizations a two-faced identity. On the one hand, organizations portray themselves as ‘effective’ and ‘rational’, while, on the other hand, internal organizational structures turn inefficient. Scripted logics for how things are supposed to be done lead to a variety of loose couplings of components that operate under their own agenda or possess separate, overlapping, and contractional institutional logics. Organizational theorists describe *loosely coupled systems* as an effect of high levels of autonomy, which is especially prevalent in educational institutions (Weick, 1976). Loose couplings describe the weakness or absence of control, influence, and coordination between organizational arrangements (cf. Pajak & Green, 2003). Different levels and branches of the organization are only loosely connected and what goes on in one sub-division does not necessarily pertain to the arrangements in others. This has both positive and negative implications. Loose couplings denote a lack of compliance between formal structures, i.e., goals, decisions, plans and lines of authority, on the one hand, and work processes and results among faculties on the other (Paulsen, 2011). However, it is precisely within loosely coupled systems that MOOC entrepreneurs find a room for maneuver, occupy their position to engage in MOOC activities and practices and even innovate and transform pedagogy.

In this regard, it makes sense to apply certain concepts from institutional entrepreneurship to grasp how MOOC entrepreneurs maneuver and engage in and across spaces situated in loose couplings. Institutional Entrepreneurship is foremost associated with the work of DiMaggio (1988), who defined institutional entrepreneurs as actors who mobilize resources with the intent to initiate changes that contribute to transforming existing institutions. Institutional entrepreneurship has also been defined as “activities of actors who have an interest in particular arrangements and who leverage resources to create new institutions or to transform existing ones” (Maguire et al., 2004, p. 657). Besides such general definitions, institutional entrepreneurs can perhaps be better understood as *change agents* who deliberately attempt to bring about change (Battilana et al., 2009), implying an agency and empowerment perspective of the actor. A major theme, which is analyzed from different perspectives in the research on institutional entrepreneurship, is to define who and what characterizes an institutional entrepreneur and what types of processes they engage in (Hardy & Maguire, 2008). Obviously, institutional entrepreneurs are somewhat engaged in activities and practices where they interact with more powerful actors, meaning that the analysis of power and legitimacy is a subtext in these research contributions (e.g.: Fortwengel & Jackson, 2016; Garud et al., 2002; Greenwood & Suddaby, 2006; Heiskanen et al., 2019; Hu et al., 2016; Maguire et al., 2004; Pacheco et al., 2010; Santos & Eisenhardt, 2009; Szabó, 2017; Tracey et al., 2011). This aspect involves organizational researchers, who turn to Bourdieu’s (1984, 1986) concept of *social fields*. Bourdieu’s understanding of social fields, which has been previously defined, enables organizational researchers to study power relations and also to grasp how change agents try to bring about change or are shaped by other forces in their attempt at organizational change. A social field can be claimed to be a dynamic model for explanation, which shows how actors interact in changing social contexts.

Bourdieu’s concept of a social field is however a broad approach, meaning that we need to delineate our analytical scope and explain which concepts we use in the data analysis to come. We are foremost interested in using the concept of *subject position*, a concept closely related to social fields. The concept will be used in two ways. Our first application of the subject position is to understand which *position MOOC entrepreneurs have in a social field*. In fact, subject positioning allows actors to position themselves in different types of relations (such as power relations), representing an approach to account for pace, time and contexts. In institutional entrepreneurship, organizational theorists have employed *subject position* to establish what characterizes an entrepreneur, meaning that entrepreneurs can be viewed as a product of a field and then either fail or succeed in exercising power. Hardy and Maguire (2008) argue that entrepreneurs do not necessarily take one singular position, as they can occupy both central or peripheral positions.

Our second application of Bourdieu’s concept involves focusing on the *interpretive struggles* that MOOC entrepreneurs experience when they try to initiate changes in the social fields that they engage in (Hardy & Maguire, 2008). This aspect surfaces when MOOC entrepreneurs interact with stakeholders or encounter the institutional agency of powerful actors who do not share the same understanding of, for example, a MOOC. The two approaches will guide us in the data analysis.

## Research Focus and Research Questions

In this study, our focus is on entrepreneurial activities following the emergence of the MOOC phenomenon. This development has the capacity to influence pedagogy, policy, and strategy in HEIs worldwide (Zhu et al., 2020). We use New Institutional theory to explore the *subject position* of Norwegian MOOC entrepreneurs and account for their *interpretive struggles*, which is marginally represented in the research literature (Langseth et al., 2022; Tungesvik, 2021). We use subject positioning to explain what happens in loose coupling, and account for struggles where power is unequally distributed. In their pockets of innovation, MOOC entrepreneurs are generally not formally challenged, yet in their encounter with more influential stakeholders and logic they struggle to legitimize their ideas in the more formal institutional arrangements. Our overall goal is to show how entrepreneurs experience and engage in complex organizational processes when attempting to promote MOOCs in Norwegian HEIs.

Norway is currently one of the most digitized countries within the OECD area, something which demonstrates Norway’s digital maturity and readiness for educational change (OECD, 2017). The Norwegian government’s strategy for digital competence development (KD, 2017–2021) emphasizes the need to strengthen the use of ICT in the entire educational system to prepare students for working life. Lessons learned from this study may therefore be interesting to a wider audience.

In fall 2020, we interviewed employees from different Norwegian HEIs who had been actively involved in the development of MOOCs since the early 2010s. We focused on the adoption of MOOCs to explore how the informants occupied the subject position of MOOC entrepreneurs through actions and practices that supported that role. We also intended to describe how they negotiated their belonging to the subject position as MOOC entrepreneurs by mobilizing resources. The following research questions guided our analysis:

1. What characterizes MOOC entrepreneurial activities and practices in the Norwegian sample?
2. What experiences do MOOC entrepreneurs draw from their engagement in educational change?
3. How can transformative models describe digitalization processes in higher education?

## Method

As we have already pointed out, entrepreneurial activities take place in various social fields within and across institutions. A good deal of knowledge about digital entrepreneurship in the social fields of HEIs is still vague and open to exploration. This calls for a qualitative research methodology and an inductive analytical strategy.

To approach our research topic, we decided to interview informants who were involved in MOOC production and support. The material was then transcribed and analyzed based on an inductive and open-ended approach. This means that the findings were established based on the transcribed interviews and what the informants told us about their activities, in this case MOOC entrepreneurship. Such inductive strategies are essential to researchers from several qualitative traditions, e.g., Grounded theory (Corbin & Strauss, 2015; Creswell & Plano Clark, 2011; Glaser & Strauss, 1967) or in this case Thematic analysis (Attride-Stirling, 2001; Braun & Clark, 2006). Thematic analysis is a systematic approach to reading text, e.g., interview data, and adding relevant themes or categories to make sense of the material. We describe our approach in more detail below.

### The Sample

To avoid researchers being overwhelmed by the data, qualitative methods limit the number of informants. We found that ten informants would be a manageable sample and selected these from ten institutions based on their visibility in the Norwegian MOOC landscape. Obviously, we realize that this sample cannot represent all shades of MOOC initiatives in Norway. Altogether, there are more than sixty HEIs in Norway (37 public and 24 private). Our sample comprises ten of these, but also eight out of the ten largest.

Furthermore, our method of selecting informants may also be described as purposeful sampling (Patton, 2002). Purposeful sampling is a widely used technique in qualitative research to identify and select information-rich samples “whose study will illuminate the questions under study” (Patton, 2002: 273). In this study, we chose institutions with merit in the MOOC landscape, and identified and spoke to informants who stood out locally as active initiators and contributors to MOOC initiatives. To identify these informants, we asked leading personnel in the selected institutions to put us in contact with possible candidates.

A criterion for selecting the informants was also that their involvement in MOOC-technology, production, and support had been going on for several years. We finally selected ten informants (N = 10), both male (n = 8) and female n = 2). Even if these individuals usually worked with other colleagues, we still limited the number of informants to one informant from each institution.

Strategic sampling, of course, impedes statistical generalizations, but we argue that this sample will let us explore and pin-point essential processes in Norwegian MOOC developments. That said, we should also mention that the same set of data was supplemented with an additional sample and used in a previous study (Langseth et al., 2022) where the focus was on support units in digital transformation.

### The Interviews

The interviews followed a preplanned interview guide with open questions. Examples from the study could be “Could you describe your MOOC activities?” or “What stoppers did you encounter on the way?”, “What is the current situation for MOOCs in your organization?” The main goal for this line of questioning was to let the informants describe their activities as freely as possible without having our questions as limitations. We wanted to know about the activities and variations in the different initiatives and hoped that the open-ended questions would elicit new knowledge in the field of digital entrepreneurship in HEIs. On the downside, however, we could not plan many of the follow-up questions or clarifications in advance. Each interview lasted about 60–70 min.

Due to the restrictions pertaining to the COVID pandemic, the number of geographically spread institutions, long distances, and potentially high travel cost, we conducted the interviews online on ZOOM. This turned out to work very well and we established good contact with our informants, who were familiar with working online and seemed comfortable during the interviews. We taped each interview with audio and video according to consent from the informants and had the audio recordings transcribed by a third party.

The interviews were transcribed as *clean copies*, i.e., non-verbatim transcripts that take out any repetitive or redundant expressions without changing the context or meaning of the conversation. The transcripts, each comprising 10–15 pages, were imported into NVivo, which was the main analytical tool. NVivo is mainly a tool for text analysis, and we made no further use of the video recordings.

The researchers and informants in this study all have Norwegian as their first language and all the interviews were conducted in Norwegian. The initial analysis was based on the Norwegian transcripts. Quotes that would illustrate the most important findings were selected during the analytical process and translated into English. The translations were done manually, and to ensure validity of the translations we took care to preserve as much as possible of the original meaning.

In the transcripts and the selected quotes, we did also not try to recreate or represent the explorative speech (Barnes, 1992) or the hesitations that are so typical for this kind of conversation. Explorative speech is a process that helps the informants develop and express their thoughts and ideas, and it often comes out as hesitations or incoherent sentences. Rather, we chose to condense the quotes and to focus on their essential meaning. In the translations we also focused on idiomatically correct English. In our opinion this does not jeopardize the validity of the quotations, nor the translations. To maintain the informants’ anonymity, we have avoided their names and affiliations in our presentation of the data. To further protect the informants’ identity in a small country, such as Norway, we have also avoided any additional descriptions fit to identify them. The main purpose of the selected quotes is to strengthen the transparency of the analysis by giving the informants a voice.

### The Analysis

NVivo helped us organize the data into categories, to better see the overarching patterns in the material and to retrieve and review the information for further consideration, comparison and analyses.

Thematic Analysis (Attride-Stirling, 2001; Braun & Clark, 2006) explains the main purpose of coding as a process where the researcher identifies significant themes from the interviews. The approach encourages researchers to examine and record patterns or themes across the material that describe the phenomena at play. In our analysis, inspired by this approach, such themes became categories for further study. Every interview was read several times to get a feeling of the main stories. Hence, based on the information that the informants provided, we carefully developed the codes that would help us make sense of the material. In the process, we looked for both general patterns and idiosyncrasies. Throughout the writing process the codes were further scrutinized, and suggestions from one author were discussed with the others to maintain the validity of the analyses. The research questions listed above were also developed further in a dialectic process with the data inspired by the constant comparative method (Glaser & Strauss, 1967). Refined research questions and codes helped us to further structure the rather large material into sub-categories and categories that would describe the data on a more generalized level. Table 1 gives a more detailed account of the analytical process and relationship between quotations, codes, sub-categories and categories.

**Table 1:** Categories resulting from the data analysis

Quotes from informants (N = 10)	Codes	Sub-categories	Categories
*“It was relatively new with MOOCs, and we started thinking, what could have been exciting here.”* *-* *“Curiosity driven, we have a responsibility to find out what is available and what relevance it will have for our own institution.”* *-* *“Canvas has not necessarily been available for external students. That is why we started with Open edX, which became a project in addition to other kinds of work”.* *-* *“(we collaborate with) active, innovative professionals who want to or work passionately for their profession. Many of them want to try out new things (MOOC)”*	New with MOOCs. What could be exciting? - Curiosity driven responsibility. - Started with Open edX for external students. - Collaborate with faculties to try new things.	Inspiration from abroad. - Curiosity driven. - MOOC platforms. Student centered. - MOOCs and micro-credentials.	**Digital transformative agency**
*” They (faculties) found that they wanted the introduction to the course to be online and I wanted to test out Open edX”.* *-* *” We found a group of faculties that was interested in making a MOOC, who were excited about it.”* *-* *” It is more like an exploration, this kind of work is fun, you do not know where you are going, what direction it takes and how to do it.”* *-* *” I think it has been a success that they have managed to produce the courses in a way that has made them attractive. (…) It has been such an extremely positive force.”*	We wanted to try an online course on Open edX. - Faculties, who were interested in making a MOOC. - We do not know where we are going and how to do it. - The production of an attractive course is an extremely positive force.	Transdisciplinary collaboration. - Team of complementary competences. - Explorative nature. - Tangible results.	**Hands-on experiences.**
*“The first online course was in 2006(Moodle). We were early adopters of distance education.* *-* *“We started as a project (Open edX) in 2013.”* *-* *“At that time (2014), we tried to partner with Coursera, and it was pretty clear that they were not interested in us.”* *-* *“We started a process with FutureLearn in 2013.”*	Took distance education online in 2006. - Started as a edX project in 2013. - Tried to partner with Coursera in 2014. FutureLearn in 2013.	Early adopters. - Various open platforms and MOOCs.	**Timeliness.**
*“The rector made the decision and funding initially came from the rector.”* *-* *“Now (2020), you will have to land a future in terms of the contract and the level of activity. It is strategic work on how to use that opportunity to a greater extent and bring it into the fold of the institution, rather than it being a hobby project.”*	Early funding from management. - Have to land a future, not a hobby project.	No clear strategic decisions.	**Feedback and funding.**
*“It is the management that has stepped in.* *it has been a process. We are, in a way, on the organizational chart. We don’t have our own box, and there are several administrative lines and several bosses who are above the people here. “* *-* *“The challenge probably lies in the fact that they have too little contact with higher levels, i.e., the pro-rector level. “* *-* *“Then there was a dispute. There was a different innovation process with a different logic, namely the implementation of (mentions LMS). The study administration should come in and manage this dispute. And there you have a history of the important environments for digitalization? “* *-* *“Everyone knows about it (the open platform), but it’s somehow not like: now we have to make an effort to put it into use. Canvas is sort of what everyone thinks is what we should use. “* *-* *“In the Nordic countries, we could have had our own platform. Something of our own? “* *-* *“It is part of the societal mission of a university to contribute to open knowledge in society. In the same way that research is open, teaching should also be open. And then, you cannot have a closed LMS. “*	In a way on the organizational chart. - Too little contact with higher levels. - Dispute over digitalization based on different logics. - Have to make an effort to use open platform. - A Nordic MOOC platform? - Societal mission to contribute to open knowledge.	Top-down initiative with many stakeholders. - Communication is limited. - Different logics at play. - Limited use of knowledge and skills. - Platforms suitable for mission.	**Institutional roles and locations.**

### Validity

Inductive analysis is very much based on the researchers’ experience and background. This means that the researcher can in fact render the validity of the research vulnerable. Therefore, it is frequently recommended that the coding process is done by two or more researchers (cf. Charmaz & Belgrave, 2012). It is also recommended that the researchers have different backgrounds, as we have in this study (Sweeny et al. 2012). As previously mentioned, we worked together to analyze the data and would take turns suggesting preliminary codes and themes. Throughout the coding and writing process these codes would be discussed and further developed by the research team. Discussions about the analyses and the writing of the text could be both in person and online. We rarely had major disagreements on how to understand the data and any difference in opinion would be amicably resolved. This kind of large-scale agreement throughout the process suggests a high degree of intercoder reliability (cf. O’Connor & Jofe, 2020).

Even so, no analysis can be completely balanced, but we argue that such intercoder reliability combined with the transparency stemming from detailed descriptions of the data, indeed strengthens the validity of our findings. We have not made any specific attempt to calculate the intercoder reliability, but we still contend that our coding strategy increases the internal validity of the data. Internal validity refers to the accuracy of the data analyses (Kolb, 2012), as the external validity focuses on the areas of reliability and generalization. Kennedy (1979) points out how comparisons with other cases resemble how doctors or lawyers compare their cases with previous cases described in medical journals or court records. Kennedy contends that generalizations from one case to the next can be made when relevant stakeholders recognize enough similarities. Hence, the relevance of the research is mostly up to the reader.

The research project has been approved by the National Centre for the Handling of Research Data in Norway. The researchers involved in the project have been active participants in developing MOOCs in Norway and had a central role in the NTNU Drive project, which was financed by the university to enhance NTNU’s digital visibility and activities online, especially related to the production of local, national, and global MOOCs. We all have previous publications on the subject related to this project.

## Findings

In our data analysis, we used Institutional Entrepreneurship theory to explore how the informants (N = 10) occupied social fields and negotiated their subject position. Our findings demonstrate how the informants positioned themselves in two overreaching themes: (1) *MOOC entrepreneurial activities and practices*, where they functioned as change agents in pockets of innovation characterized by a room for maneuver and (2) *MOOC entrepreneurial resource mobilization*, where their room for maneuver was defined by other more powerful actors (at macro-levels) in the institutions and where they negotiated their position and ended up in interpretive struggles.

### MOOC Entrepreneurial Activities

The first theme in our data analysis establishes how actors occupy the *subject position of* MOOC entrepreneurs in teaching and learning at micro- and meso-levels in their organizations. To examine what characterized the MOOC entrepreneurs, we looked for common factors in the way they initiated new activities and practices. We found three categories: digital *transformative agency, hands-on experience and timeliness* embedded in the initial stages, mainly from 2013 to 2015 onwards.

#### Digital Transformative Agency

The first category was *digital transformative agency*, which we identified as a process where MOOC entrepreneurs, either individually or as part of a group, explored the educational value of MOOCs and MOOC platforms. The informants described themselves as among the first to take a serious interest in MOOCs and open platforms in Norway. Their individual interest originated in different areas, ranging from technology to digital pedagogy and educational strategies. They teamed up with a common goal to develop the MOOC phenomenon and support other faculties to cater for equity and quality in student learning in their local context.

*For many students, the alternative to online learning is no studies on campus, it is no teaching and learning at all. So, if you want an offer for these students, you must go online.*

To describe their source of inspiration most informants pointed *abroad* to George Siemens, Stephen Downes and Connectivism (Siemens, 2004) and the first connectivist cMOOC in 2008, which was centered around the learner and networked learning (Downes, 2020). One informant also described the process of retrieving new insights as both an opportunity and a responsibility:

*In a way, we have a responsibility to find out what is available and what relevance it will have for our own institution, and we have always had a kind of openness to be able to explore.*

Informants further reported being inspired by asynchronous courses online, the so called xMOOCs, which were centered around a professor and based on more traditional university courses, mainly formed by leading HEIs, such as Perth, EPFL, MIT, Harvard, Stanford and the Open University (Langseth et al., 2018). Several informants had visited these institutions and a few informants had explored the possibilities of partnering with open platform providers, such as Coursera and FutureLearn.

*I’ve been working on these courses from 2015, when we entered into an agreement with FutureLearn. I think that, at the time, they (leadership) put emphasis on the fact that they wanted a European supplier, and the things that concerned storage in the cloud and data security.*

Informants were driven by *technological advances*, as they described a particular interest in exploring Open-Source technologies and MOOC-platforms. Partnering with global platform providers was, however, a costly and time-consuming decision that had to be made at higher strategic levels in the organizations. Hence, some informants turned to open-source platforms, such as edX and Moodle. Hosting the platform on existing servers in the IT-department was described as challenging, and some had turned to third parties for support (cf. SIKT^1^). Others used Canvas Open, while some were limited to the LMS, which required students to register to get access to the course content, in their institution.

*I am probably the only one who administers Open edX here at the university. It has not been introduced to the same extent as Canvas in any way. And it has been a long way to go with the IT department to try to introduce another system. They don’t quite see the reason for it and believe that Canvas should solve the challenges with teaching.*

*Our official platform is Canvas (closed) and we have not been able to use it for course participants, because only those who take credits and exams have access to it. So then, we had to find a platform that we could use, and then we started with (mentions open platform). We can use the platform for both small and large groups of external students.*

Informants also took an interest in *learning design*. As expressed by one informant: “*One thing is the technological innovation, but it is the pedagogical innovation that often becomes so difficult.”* When working on learning design, informants generally described their entrepreneurial activity as driven by curiosity and willingness to take a «trust leap» (Botsman, 2017) beyond their comfort zone, to push boundaries and gain new insights for something bigger than themselves. Their idea of learning design was centered around multimodal texts and social learning, as well as variation in the pedagogical approach:

*You must have a learning design where you have a range of offers, where you have the information, in both text and video form, and where you also integrate social learning activities.*

One informant expressed, for example, the excitement about the potential in MOOCs and how their success thinking could be important for students, as compared to what is available in their ordinary studies.

*It was relatively new with MOOCs, and we started thinking what could have been exciting here. And I’ve always had that idea that success thinking is something important for students, that is, something that you don’t really get in your studies and do a bit as a sidetrack.*

The informants were primarily interested in learning to develop the capacity to support faculty and thereby, to provide the best learning experience for students in MOOCs. In the process, informants sometimes experienced controversies when collaborating on learning design, as some faculty members that were new to MOOCs, seemed to struggle to adjust their pedagogical practice:

*I think they are, at least initially, very “I know best”. They are, in a way, not open to changing anything. (…) They start making “patchwork quilts” for online studies instead of starting all over again.*

Several informants were also inspired by MOOCs they had either taken or contributed to in international contexts, such as in projects funded by the European Commission. One informant described these processes as: “scouting for new insights” and “*in a way, ask what this is and how to work with it?”.*

An overall observation was that the source of inspiration for change was based on new information and some experience with MOOCs. In the process, they also reflected and acted upon this knowledge in their own local contexts. The white paper MOOCs for Norway (NOU, 2014: 5) also served as a welcoming and positive confirmation of the informants’ ongoing initiative at the time. A general finding was that initial MOOC activities and practices took place among faculty and staff at lower levels in the institutions. There was not much enthusiasm about visionary thoughts in leadership and governance documents:

*Generally speaking, I am not impressed by the level of vision in speeches and governance documents. In the world, people are already in the process of establishing a new practice and have come much further than us. I would say that most of the innovation and inspiration, the visionary thoughts and ideas take place at lower levels in the institution.*

#### Hands-on Experience

The second category that we identified was *hands-on experience*, meaning that informants were closely involved in tangible outcomes. All informants reported that they had been actively engaged in MOOC production. They had, for example, been involved in the co-production of academic MOOCs for various groups of students in Norway and abroad, and MOOCs for internal competence development directed at faculty. Some informants had also produced and run their own MOOCs, with badges and accreditation (ECTS). Examples of early MOOCs were in the fields of music, information literacy, smart digital learning, ancient cities, and addiction to gaming. The informants typically described an explorative production process, where they teamed up with other faculty, and sometimes external partners, to meet the necessary competency requirements to produce the MOOCs. To illustrate the process and the work involved, we turn to one informant, who collaborated with the university library to create a MOOC on teaching information literacy:

*They (library) were to work with information literacy teaching, and they wanted the introductory part of the course as an online solution. And I wanted to test Open edX. (…) In 2013, we started with Open edX and the construction of this course. Now, we have several thousand registered users, and the number has gradually grown because there are several subject areas at the university where our course is a work requirement. (…) So, I have put an awful lot of work in that course. (shortened)*

In the collaborative production process towards a small and tangible goal, like the creation of a first MOOC, they experienced success. Informants reported that they had achieved something of value to themselves and the university. One informant expressed their achievement as “an extremely positive force”. They had produced an attractive course with many participants and had received positive feedback from learners, which gave something back to the faculty and the professional community.

Informants also reported that faculty and management became interested and that some found MOOCs exciting, especially when they saw the results: “There is applause and a big speech”. Then afterwards “it can flatten out a bit”, suggesting that the interest was, to some extent, transient. To illustrate the level of interest and activity in these initiatives, we report from one Norwegian HEI, where the global MOOCs provided on FutureLearn (in English) and the Open edX platform (in Norwegian) had attracted some 70,000 learners in 4 years from 2017 to 2020.

A general observation from our data, which has also been discussed in a previous preliminary study (Langseth et al., 2021), is that the tangible results coming out of their activities in the local contexts can be characterized in four typologies. Here, the informants occupied the *subject position* of MOOC entrepreneurs while framing their educational activities and practices on (1) the choice of target groups, (2) the language of instruction (Norwegian or English), and (3) the kind of platforms that were available to faculties (open platform and/or LMS) in the HEIs. The four typologies resulting from these hands-on experiences were divided into four areas: global MOOCs, national MOOCs, special purpose MOOCs and closed online courses (LMS).

1. *Global MOOCs* tended to be micro credentials of general and specific interest with a global reach. The courses were Open Access and provided a course certificate upon completion of the course. Only informants from the larger universities reported such course offerings. Typically, the courses were in English on FutureLearn.
2. *The national MOOCs* were MOOCs of general and specific interest with a national reach. Courses were offered on open platforms with Open Access and could provide learners with a course certificate (badge) upon completion of the course. Some courses also offered ECTS, which implies that learners had to register as students at the HEI prior to taking an exam. Typically, these courses were offered in Norwegian at level 6 or 7 (bachelor or master level qualifications) on Open edX, Open Canvas or Moodle.
3. *The special purpose MOOCs* were MOOCs or micro-credentials that tended to be offered on a *by invitation only* basis, for internal competence development and professional collaboration with external partners. They were, for example, offered in obligatory pedagogical development programs at universities and mentoring programs for teacher training and nursing to strengthen the quality of students’ learning in placement. These courses tended to address institutional needs for flexible and scalable access to information and could sometimes be combined with synchronous contact points online or on campus. These courses were often provided on open platforms, e.g., Open edX, in Norwegian or English and sometimes provided successful learners with a badge upon completion of the course.
4. *The online courses on an LMS* were closed online courses with limited regional and national scalability and with more synchronous and teacher supported learning activities. They were mainly targeting Norwegian students and often supported by the Norwegian government (cf. project funding) in connection with further education and lifelong learning, for example for teachers. Typically, participants registered as students at the HEI prior to getting access to course content and an exam, which often ranged from 5 to 30 ECTS. Courses were provided on the existing learning Management system (LMS), like Canvas or Blackboard.

Some of the MOOC entrepreneurs in our study were restricted to online courses on the LMS and traditional organizational arrangements for courses and study programs in their institutions. One informant also expressed concerns about the use of the LMS as a place to “store files and folders” and advocated the need to rethink content in terms of “text, video, social learning and student engagement” in all courses in HE. Another informant stated that after having experienced working with FutureLearn, “one can base the learning design more or less on the same principles on Canvas (LMS)”. These findings suggest a transfer value of MOOC entrepreneurship on teaching and learning on campus, for example in blended learning (a mix of online and on campus teaching and learning).

#### Timeliness

The third category in our findings is *timeliness*, meaning that the MOOC-phenomenon in Norway can be seen as a *timely process* that initially followed the international advances in MOOCs on open platforms. The time span was relatively short from the early 2010s, when Connectivism, MOOCs and MOOC-platforms appeared internationally, until the early adopters in Norway had their first MOOCs up and running on an open platform that gave unlimited access to course content. By the early 2020s, the international trend had expanded considerably, as we observed that MOOCs in Norway were still at a small scale.

**Figure 1:**
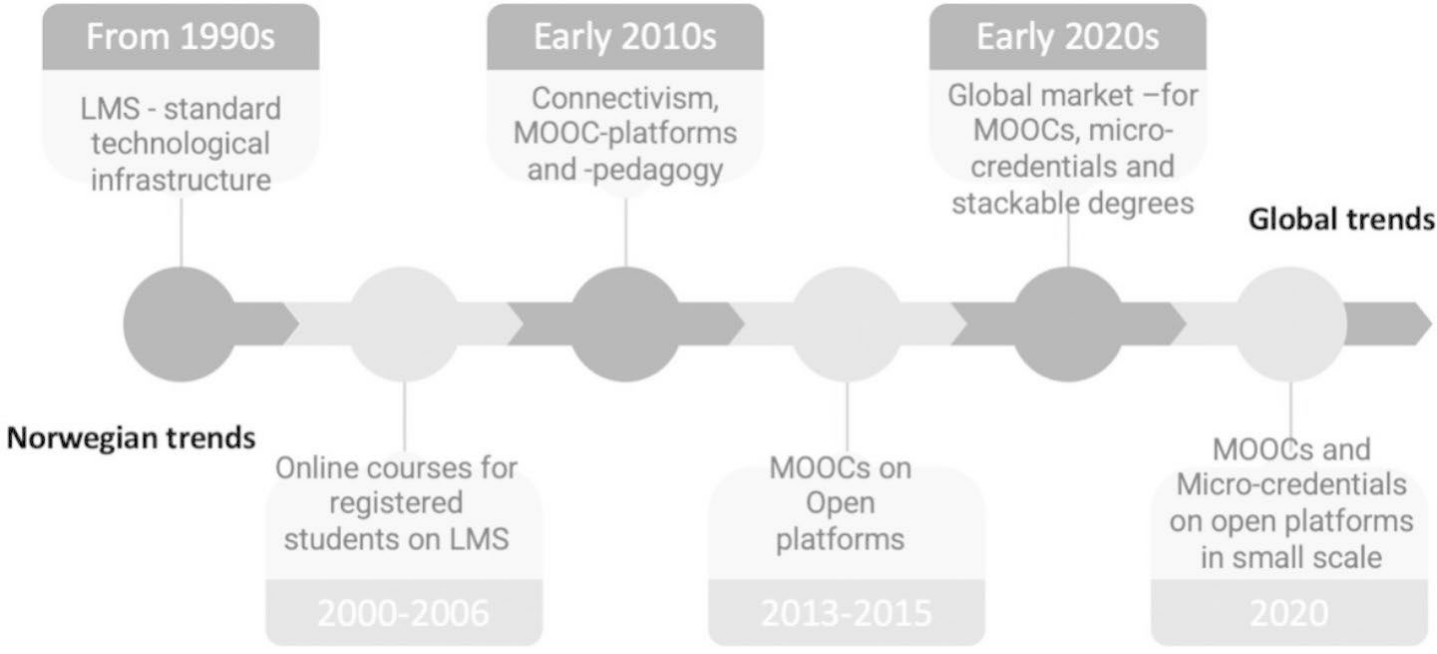
Timeline of MOOC entrepreneurial activities in Norwegian HEIs relative to global trends

As shown in Figure 1 above, we could trace the MOOC entrepreneurial initiatives in Norway on a timeline to illustrates how MOOC entrepreneurs adapted their activities and practices to the international context. For example, two informants reported that the adoption of MOOC-technology seemed to be fueled by the institutions’ long experience from correspondence by post, videocassettes and sending teachers to remote places. They used Moodle as the preferred platform when they transitioned to online education from 2006. Around 2013–2015, several informants (co-)produced MOOCs that were flexible and scalable alternatives to education on campus on open platforms, mainly on Open edX. Three informants also reported that they had partnered with FutureLearn to produce MOOCs with a global reach in agreement with the management at the time. That said, some informants lamented that they were still locked in the closed platforms (LMS) in their institutions and could not opt for open platforms. Even so, they adopted the asynchronous design principles for MOOCs in their courses, mainly on Canvas for campus. We see a shift in Norway from the first MOOC initiatives, where we could observe timely initiatives, to a second phase, where Norwegian faculties start lagging behind the global development in online course offerings.

### MOOC-entrepreneurial Resource Mobilization for Institutional Change

The second theme in our data analysis establishes the subject position of MOOC entrepreneurs in social fields governed by more formal and strategic institutional arrangements. To occupy these *fields*, entrepreneurs tried to position themselves to create change beyond random initiatives and time-stamped projects related to practice. Those who managed to successfully negotiate their position had to adapt their activities and practices to formal structures and the organizational line. Hence, they entered *interpretive struggles* about resource mobilization in terms of funding, legitimacy and strategy at higher levels in the organization. The tensions that resulted from these struggles were often related to power, as MOOC entrepreneurs engaged in institutional agency in contexts formed by other discourses in the organization.

To find what constitutes the characteristics of the MOOC-entrepreneurs in these fields, we looked for common factors in their engagement with stakeholders, leadership, and organizational strategies to instigate institutional agency through MOOCs and micro-credentials. We found two categories: *feedback* and *funding*, and *institutional roles and locations* embedded in an ongoing interpretative struggle.

#### Feedback and Funding

One main category was that of *feedback and funding*. Feedback is recognized as a driving force in teaching and learning (Hattie & Timperley, 2007) and funding constitutes a driving force in strategic initiatives, project proposals and evaluation procedures in HEIs (Diefenbach, 2009). Access to feedback and funding were a major concern for MOOC entrepreneurs.

Informants legitimized their agenda by pointing to *positive* feedback from faculties, who had co-produced their first MOOCs, and students, who had taken their MOOCs. They were backed up by relatively few leaders, who had taken an interest in their work, while tangible results were formally acknowledged and celebrated:

*People get interested and they find it (MOOCs) exciting, especially when you see the results. (…) You see that this works, and then there is applause and a great speech. Good work, well deserved.*

In contrast, when asked about how MOOC-initiatives were received by important stakeholders in the organization, informants often described the feedback they received as generally *reluctant* and sometimes ignorant, implying that some stakeholders were hesitant to funding the activity, some were not informed, and others concluded that the existing LMS was sufficient. Informants generally experienced flaws in internal communication about ongoing activities and support for MOOCs.

*Also, I think there are quite a few people who don’t know about it (MOOCs) or have heard of it at all. (…) when we took those rounds and asked for funding to be able to continue the activity, who will pay for it then? There are some who say yes to funding, but ask what is this really about? What are they going to do with it (MOOCs) anyway? What’s the deal here? Some hastily conclude that we have already got (mentions LMS).*

Constructive communication was generally considered a major challenge. One informant pointed to the challenge involved in communicating new ideas at higher levels, and even questioned the ability to do so:

*I think that maybe we have not done a good enough job of conveying this idea, to get the information out and explain it, even though we think we have done it many times and in the right forums.*

Some stakeholders also seemed to question the social mandate of equity and democratization of student learning outside the more traditional courses and study programs. They argued that there was no need to fund courses for students that they would never see again:

*Resources go to help students, who we may never see again, or who are not even students. Who are we teaching for, in a way, and is this what the university should continue with? This is kind of an entry into it (communication with leadership).*

Informants further described the need for *access to stakeholders* at higher levels to negotiate their belonging to the subject position of entrepreneurial change agents in the field. Only a few informants reported that they had access to strategic stakeholders’ time and attention in their HEIs. They also tended to be more successful. Those with limited access to stakeholders found it difficult to negotiate their position and receive funding and strategic feedback on their roles and activities. That said, many informants also experienced that leaders did not possess a *sufficient vocabulary* to discuss the logics of MOOCs and micro-credentials and the possible implications for HEI in a broader perspective in the limited time available in strategic meetings in their organizations.

Positive feedback on project proposals that resulted in external funding was common, as feedback on possible long-term outcome scenarios for these MOOC projects seemed to be less informed, organized and systematized at higher levels in the HEIs. Nevertheless, the funding that these MOOC-projects received was considered important for both MOOC entrepreneurs and management. What they offered in terms of results was often seen as additional activities on the “side-line” from the perspective of the informants. Some informants described their working conditions as a journey “from project to project”, as their organization was mainly focusing on digitalization and support in more traditional educational scenarios on campus, e.g., LMS. One informant sums up the struggle to occupy the subject position of entrepreneurial change agent in the field in the following way:

*So, you undergo an application process. First pre-qualification and then qualification, then project contract. And in all these steps, the top management is involved to show their commitment. (…) And afterwards, when it is discussed in the organization, (managers say) that these projects are not legitimized and where did this come from, and so on. The institution wanted to get hold of that money, and that activity and then they signed, but didn’t really mean it. Or yes, they do ideally, but they do not really consider what the consequences of these decisions actually are. (…) Innovation in academia is like one funeral at the time. (Shortened)*

The informants clearly struggled to position themselves in the social fields, i.e., to enter an informed discussion about what they had achieved, what the organizational outcomes could look like, and participate in forming future strategies. Their lack of opportunity to negotiate their belonging to the subject position as change agents was experienced as a lost opportunity for institutional agency and digital transformation. A main concern was that “as competence on MOOCs accumulates and spreads in positive ways in the organization”, many informants were still pondering how to “get their knowledge across”. An overall assumption was that stakeholders and management struggled to allocate time and attention to be informed about what was going on in the many entrepreneurial projects at lower levels in their institutions. Interpretative struggles based on competing logics and the “administrative manner of speaking” strengthened the complexity when MOOC entrepreneurs tried to occupy their subject position in these fields. Consequently, MOOC-entrepreneurs often received both external and internal funding, as feedback on project results was often inconclusive or put on hold in terms of the possible long-term outcomes in the HEIs.

#### Institutional Roles and Locations

A second category is the *formal organization* of *MOOC-entrepreneurial roles and locations* on the organizational chart. Such decisions were taken at higher levels in the institutions, usually without much involvement from MOOC entrepreneurs. Only a few informants had managed to occupy more powerful subject positions (Törrönen, 2001), where they had established trust and could confidentially argue the value of open platforms and instructional design in MOOCs and micro-credentials to stakeholders in the field.

Initially, MOOC-entrepreneurial *roles* represented a broad mix of faculty and administrative staff located across institutional silos and organizational arrangements. They had formed pockets of innovation with the necessary complementary competences to produce MOOCs at lower levels in the organization. Their background spanned from Interaction and Communication Technology, media production, journalism, library studies, pedagogy and various academic fields. Consequently, they were in fields spanning from the university library to technological support units for the LMS and teaching positions in their institutions. *Locating* these roles on the organizational chart was complicated and a matter of strategic concern as the activity expanded and challenged the existing administrative routines. Hence, we found three typologies in the way these MOOC-initiatives were organized over the years: random initiatives, project management, and reorganization.

- *Random initiatives* - informants reported on ad-hoc initiatives and limited funding from various sources. These entrepreneurs used their room for maneuver to explore the MOOC phenomenon within their ordinary working hours or “as a side-track”. They also found others, who were interested in MOOCs, to collaborate with.
- *Project management* - informants reported that they were supported by their institution through internally funded and various externally funded projects, often in a mix. They had been involved in numerous project proposals and MOOC projects over the years. Each project typically lasted for 3 years.
- *Reorganization* – Some informants had been relocated in formally established support units or learning hubs of sorts. In this scenario, an existing unit typically expanded and assimilated MOOC-entrepreneurs in their activities. Their room for maneuver was framed by administrative leadership and management in these support units.

Several informants reported that they had moved from project-based funding to regular funding, as the organization had been reorganized. Interestingly, we found that many of the more recently established support units had few or no academics with allocated research time. Informants often report that systematic and research-based development of MOOC initiatives in these units was rare, as participants often occupied administrative positions. A general finding was that, while the level of innovation was relatively high, systematic research on their own activities was often described as limited or non-existent. Rather, research among MOOC-entrepreneurs seemed to exist in projects, where academics had research competence and research time. Something, which is also confirmed by Tømte et al. (2020), who described one institution as responsible for nearly half of the eleven refereed articles on MOOCs by Norwegian authors. Several informants with an academic background found reorganization problematic since they would lose their research time.

As for academics who temporarily engaged in MOOC production, an overall finding was that there seemed to be little or no strategic incitement to produce MOOCs and micro-credentials in place. For example, informants referred to the lack of institutionalized arrangements on their formal work schedule when they produce and ran MOOCs. Informants specifically referred to the lack of merit or credit involved in MOOCs, which they considered hard and exciting work. Informants experienced that MOOCs still tended to be associated with further education and external funding, meaning that when the funding was over, the MOOC-courses were often not offered again. An overall finding was that the current formal organization of MOOCs for flexible and lifelong learning was still explorative and in the making.

Furthermore, informants often experienced strategic decisions concerning the more overreaching and systematic implementation of support for MOOCs as lagging at organizational levels. For example, informants repeatedly described the dissemination of their results in negative ways: *It has not been successful considering the HEI, we fail in getting the message out.* To illustrate the complexity in the point that the informants were making, we found that their activities were mainly located across organizational charts and horizontally aligned, as opposed to online and ordinary campus courses on LMS that were generally organized in pre-existing programs and where course collaboration took place in more local arrangements following vertically aligned and established practices. One informant pointed to the loose couplings between MOOC-entrepreneurial innovation and top management and higher strategic levels in the organization. Another informant experienced the lower leadership levels in the organization as more agile and ready to innovate, as the distributed approach was described as lacking in consistency at systems level. Yet another informant referred to the long-term perspective of change and the mix of excitement and frustration involved when facing resistance to change in the organization:

*No, there are no shortcuts here. It (MOOC activities) can be frustrating, but what also makes it fun, is the fact that the little work you do here challenges in a way the whole model that is safe and nice for the institutions.*

A general observation was that the timely MOOC-initiative in 2013–2015 had been quietly operating in the shadow of campus-based education, only to reappear in connection with the COVID-19 pandemic and more strategic discussions on lifelong learning and micro-credentials at higher organizational levels. Some of the HEIs in this study were, for example, involved in the European Commission action European Universities (Erasmus + KA2 projects 2020–2024) aiming at international collaboration on micro-credentials and stackable degrees in HEIs. Informants were informed about this development, but further research will establish to what extent MOOC entrepreneurial competences will contribute to shaping digital transformation and educational strategies in these contexts.

An overall picture showed that MOOC entrepreneurs tried to occupy a position where they could negotiate their role in the field of online teaching and learning. Here, they met more powerful actors that formalized and framed their activities. The tension that existed between MOOC entrepreneurs and other stakeholders was related to funding, access to technology and MOOC offerings, but also to power structures, knowledge, and belief systems in the organizations. Even so, they still believed in their mission.

## Discussion

In this part, we will discuss the two themes resulting from our analysis - *MOOC entrepreneurial activities and practices* and *MOOC entrepreneurial resource mobilization* for institutional change - and the potential for wider contributions to organizational conditions for digital transformation. A major concern is to address the implications of entrepreneurial activities and practices occurring in loosely coupled systems. This will allow us to explain how MOOC entrepreneurs occupy subject positions, and the challenges they experience when attempting to initiate changes in social fields within HEIs.

We will outline our discussion in four parts. First, we highlight and list the study’s research contributions and connect them to the previously outlined research stream. Second, we discuss how MOOC entrepreneurs contribute to innovate educational practices in the Norwegian HEI. Third, we theorize project management and the concept of pocket of innovation, while we outline how MOOC entrepreneurs contribute to digital transformation and a paradigm shift in HEIs.

### Summary of Findings – Interpretative Struggles

We found that the organizational conditions for realizing MOOC initiatives in Norway were complex and still in the making. That said, MOOC entrepreneurs tended to agree on some major concerns, which also sum up our findings.

One major concern was access to *relevant technological infrastructure*, such as open platforms where online courses are easily accessible for those who make them and those who want to learn online. In fact, while European countries have national MOOC platforms, Norway has none. Only a few universities used open-source platforms, such as Open edX, with the same setup as global MOOC platforms. Hence, many online courses were promoted as MOOCs or asynchronous online courses, while they were in fact hosted on local LMS. These university courses were credit-bearing, and students were subject to formal competence assessment (cf. K-12 or bachelor’s degree) before being admitted to the courses. Moreover, only a few universities had contracts with global MOOC platform providers; the Norwegian University of Science and Technology (NTNU), the University of Oslo, and the University of Bergen, where they offered courses on FutureLearn. An overall finding was that Norwegian HEIs were hesitant to transition to fully online courses and study programs on MOOC platforms. One reason seemed to be related to little knowledge about the global development of MOOCs and micro-credentials and a firm belief in campus-based education for their students (cf. LMS).

A second concern was that there was little or no *incitement* to produce MOOCs and Micro-credentials in place, and especially the lack of credit or merit involved in MOOCs. Faculty who received funding to produce a MOOC experienced for example that their courses were discontinued without further discussion when the project period ended. Also, there was no system for the calculation of the workload involved in producing and running a MOOC. Entrepreneurial attempts at developing a business model for MOOCs were left unfinished. Hence, MOOCs were mainly modelled and budgeted on administrative systems for campus courses, which faculty seemed to question in terms of workload and merit, especially after their first experience with MOOC production. We see these findings in connection to the low priority of MOOCs on the strategic agenda and the distributed nature of the entrepreneurial activities across institutional silos, which involved many stakeholders at lower levels in the organizations.

A third concern was that of *feedback and funding*. MOOCs were often administered and funded in time-stamped projects with a tangible and measurable result, a model based on New Public Management. Hence, human competences and skills could easily disappear when the funding dried out. One consequence of project management was that issues related to technological, legal, systemic and strategic change were difficult to land in the timeframe of a project. The lack of leadership engagement and informed feedback on the project results in some organizations contributed to this concern.

A fourth concern was that of *effective communication*. MOOC entrepreneurs often struggled to translate their innovative ideas into an administrative language that resonated with more important stakeholders. Some simply felt that their ideas were not understood, and that their knowledge was, to some extent, ignored or overseen at higher levels in the organizations. The finding aligns with Weick’s argument that the transition from one social field to another represent two distinct systems, where administrative leaders form strategies and policy and administer faculties and staff, and where faculties have the responsibility for curriculums, students, teaching and learning assessment (Weick, 1976; 2001). Effective communication is related to knowledge about the social field, the language in use, and the capacity to engage in discourses where opposing perspectives are considered (Sjøvold, 2022). Only a few informants in our study had sufficient access to stakeholders’ time and attention to contribute to institutional agency in the field, something which may be explained as an effect of loose couplings (Weick, 1976; 2001). Hence, the slow development of MOOCs in Norway may be traced back to how communication across institutional arrangements flows.

A last concern, which we find particularly troublesome, is that very few MOOC entrepreneurs had *research capacity* and allocated *research time*. In fact, those who were located or relocated in the administrative line tended not to carry out research on their activities. Without substantial research that can inform a systematic development of MOOCs, the field will surely not be taken seriously. In many HEIs in Norway, MOOCs therefore seem to be perceived as a technological and administrative responsibility, rather than a research field in its own right.

Consequently, Norwegian MOOC entrepreneurs mainly operated within *pockets of innovation*, which we have come to define as a relatively small groups of early adopters with complementary competences who are working towards a common goal, characterized by the implementation of new ways of thinking, acting and organizing. The driving force behind their activity was the belief in open access to education and open platforms to cater for equality and equity in student and adult learning. Hence, the development of new technologies and ways of synchronous and asynchronous teaching and learning online propelled their practices. In the following, and as we answer our research questions, we will first concretize what comes out of these entrepreneurial activities before we address the interpretive struggles involved.

### The MOOC Entrepreneurial Contribution to Norwegian HEIs

The first theme in our data analysis addressed an important question raised in institutional entrepreneurship; who are entrepreneurs and what characterizes them, and moreover, how can theoretical constructs be applied to identify them? For example, entrepreneurs can easily be distinguished by analyzing personal identities such as traits and biographical data, or, in our case, by examining the subject position they occupy in a social field, which implies where they are located and what kind of practices and activities they engage in. When we apply relevant theory, MOOC entrepreneurs occupy multiple positions and engage in complex activities and practices.

To concretize, these MOOC entrepreneurs made open platforms available to faculty. They developed knowledge about the learning design in MOOCs - the use of text and video, automated feedback, discussion forums, online exams etc., and how these elements could be combined to create a better learning experience for a wide range of learners, including ordinary students, faculty and others. They also explored how the learning experience in asynchronous MOOCs and micro-credentials could be enhanced by introducing synchronous elements like meetings, groupwork and feedback online. The personal theories (Robinson, 2017) that guided their practice were mainly based on Social Constructivism and Connectivism. The results of their activities in terms of MOOCs were relatively successful and diverse. Their tangible outcomes contributed to change in the social fields, where they typically occupied the position of entrepreneurs, sought results, and wanted to see the effect of their achievement in the organization.

Collaboration among the MOOC entrepreneurs mainly happened in teams within their own HEIs, where they formed (in)formal networks across institutional silos. That said, there were other, more limited, attempts at networking. Entrepreneurs networked across HEIs to establish an open platform at national levels, involving government agencies. They networked to make sense of the global platforms and exchange experiences related to technical, judicial, and strategic questions under debate in their organizations. Some faculty members also connected with international colleagues and universities that fronted MOOC research and development. Only a limited number of entrepreneurs, with allocated research time, carried out collaborative research on their MOOC activities. A common denominator was that these networking efforts contributed to developing the MOOC concept in social fields in their organizations. A prerequisite for their entrepreneurial activities was a room for maneuver to test out new concepts and ideas outside established organizational arrangements. That said, networking had little strategic effect at higher and national levels.

In short, MOOC entrepreneurs engaged in initiatives characterized by change agency, hands-on experiences, and timeliness. Change agency was rooted in areas of personal interest and converted into hands-on MOOC-production in a timely process influenced by pedagogical advances, technological opportunities, and collaboration.

### Pockets of Innovation in Loosely Coupled Systems

The second theme in our data analysis raised another important matter that is discussed in institutional entrepreneurship, i.e., the ongoing struggles entrepreneurs experience when engaging with more powerful institutional stakeholders. Such struggles often emerge when entrepreneurs attempt to initiate change and experience that their results do not lead to desirable outcomes. Entrepreneurs can express struggles in different ways, related to proper recognition and shared logics, in their attempt at change. In our case, MOOC entrepreneurs tried to perform entrepreneurial practices and activities in loosely coupled systems, where they were situated in pockets of innovation. From these pockets, MOOC entrepreneurs entered interpretative struggles, as they sought to expand and legitimize their activities in social fields at higher levels in their organizations. That said, we will further theorize *pockets of innovation* and describe a model that can potentially explain the organizational dynamics and why MOOC entrepreneurs struggle to change the educational practices.

To concretize, the tangible results coming out these pockets of innovation (cf. Chapter 6.1), competed with other initiatives, and sometimes met opposition from other stakeholders. Moreover, MOOC entrepreneurship also seemed to be filled with controversy. On the one hand, MOOC entrepreneurs entered ”a bit of a fight” over, material resources in terms of funding and allocated time to continue and possibly expand their activities. Faculty typically “fought” for funding to position themselves in the field, while staff typically “fought” to spend time on MOOC activities, not on other pressing administrative tasks. To succeed they needed attention and acceptance from the leadership and material resources to expand their room for maneuver. This room for maneuver was mainly possible because the institutions shared the characteristics of a *loosely coupled system* (Weick, 1976; 2001) According to Weick (1976: 5), loose couplings can be understood as “a situation in which elements are responsive but retain evidence of separateness and identity”. The concept explains the weakness or even absence of control, influence, coordination or interaction between MOOC entrepreneurs and other important stakeholders in the organization.

On the other hand, MOOC entrepreneurs displayed a sense of *urgency* in their current MOOC entrepreneurial roles. When reflecting on new educational pathways in online scenarios, they stated the need to “take a next step forward”, meaning that they wanted to expand and formalize their activities on the organizational chart. In this context, we found that entrepreneurs’ values and beliefs concurred strongly with disruptive ideas about digital education and institutional practices. Usually, these ideas were different from existing practices, strategies, organizational goals and leadership mind-sets. Yet, when institutions measured excellence, this was against different standards criteria and logics (Lillejord et al., 2017). This could also lead to alienation and burnout among MOOC entrepreneurs.

The MOOC entrepreneurs also reported how the move from entrepreneurial innovation to a more established and legitimized practice could be challenging. While *project management* was a normal routine, reorganization was a strategy to remove physical and legal barriers more permanently. The latter is expected to strengthen collaboration across institutional silos (Lillejord et al., 2017). In project management, the lack of continuity was obvious. Yet, MOOC entrepreneurs who were reorganized, still faced conflicts over material resources and had limited room for maneuver. One illustrative example is the open platforms that often had a loose institutional anchoring and legitimacy after reorganization. The Open edX entrepreneurs, who networked and pushed for national action, described their efforts as unsuccessful, others reported on a gap in the organizational arrangements for MOOCs. As opposed to scientific research, where institutions recognize strategic support for start-ups and funding for long-term outcomes, educational development related to MOOCs tended to capture innovations in a series of projects and dissemination strategies (e.g., project evaluations, reports, and presentations) in the organizations. These strategies seemed to have limited and slow impact on the move to establish MOOCs as part of the institutional arrangements. Moreover, because reorganization often involved administrative staff only and was mainly administered in what Bygstad et al. (2022) describe as another form of digitalization, they still faced similar challenges after reorganization.

**Figure 2:**
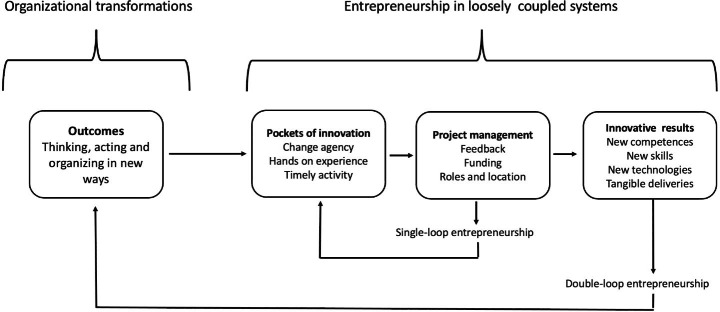
The move from single-loop to double-loop entrepreneurship in project management

Concepts from organizational theory may be useful to explain why Norwegian HEIs did not envision MOOC initiatives as disruptive and a research field in its own right. *Single loop learning*, as defined by Argyris and Schön (1978), generally describes problem-solving to improve the system as it already exists. A significant shift of perspective or practices only occurs when organizations engage in *double loop learning*, where former practices are questioned and sometimes replaced. We found that MOOC entrepreneurs mostly operated in single loop learning scenarios, even though they worked with new and disruptive ideas. In contrast to other HEIs worldwide, these ideas were rarely picked up outside the pockets of innovation. This approach constituted what can be labelled *single loop entrepreneurship* (see Fig. 2), meaning that project results rarely resulted in outcomes that lead to transformation in education in the organization.

Figure 2 above illustrates and expands the concept of entrepreneurship in loosely coupled systems at project levels. The model explains why change is difficult to achieve, also when funding is provided. When there was limited feedback on the further development of the project results, the entrepreneurs were stuck in their pocket of innovation, with limited possibilities to develop their roles and position in the organization. The result was single loop entrepreneurship, and they started all over again in another project. In contrast, when there was positive feedback from management, successful project results, in terms of tangible deliveries, new competences and skills, could lead to outcomes in the organization. These outcomes were related to organizational transformation and involved thinking, acting and organizing in new ways. *Double loop entrepreneurship*, where established practices in fact change, may start when the organization pays attention to pockets of innovations. Important stakeholders at higher levels are leaders who learn (Fullan, 2021). They make informed judgement of innovative project results and acknowledge the value of human resources, new competences, new technologies and tangible deliveries.

One reason for the development not going beyond single-loop entrepreneurship seemed to be the language barriers that apparently existed between entrepreneurs and university leaders and management. In institutional entrepreneurship theory, lack of translator competence (Furu et al., 2014), explains why entrepreneurs battle with the more powerful actors in the organization (Battilana et al., 2009). The informants often experienced that they were powerless in bringing their messages across to important stakeholders in other parts of the organization. The informants used a digitally informed language to promote their results and the wider implications for their organization, a language that often did not resonate with the important stakeholders. The development of translator competence to ease communication and help MOOC entrepreneurs position themselves in an administrative language in the organization was limited.

Another reason for single loop entrepreneurship was the lack of research on ongoing activities. We found that research and theory development related to MOOC production was often not a priority and not sufficiently supported. In contrast to entrepreneurial activities in other fields in HE (Feld & Hathaway, 2020), MOOC entrepreneurs developed the innovative ideas, but they lacked the necessary resources to disseminate research-based initiatives outside their pockets of innovation. Research that could inform viable business models for MOOCs was, for example, in most cases, absent.

In Figure 2 double-loop entrepreneurship is a central driver for change. Informed leaders are a prerequisite for the move beyond single-loop entrepreneurship to a stage where the urgency and timeliness of double-loop entrepreneurship will benefit the whole organization. In the model, management is associated with single-loop entrepreneurship, as leadership is associated with double-loop learning. We contend that there were obvious loose couplings at several levels in the organization and that loose couplings, in addition to opening a room for maneuver, also came with a down-side. Loose couplings give room for pockets of innovation, but they bring little capacity for support and reinforcement by the management when new practices are tested out and implemented at lower levels. Hence, MOOC entrepreneurial contributions in and across institutional arrangements were largely unresolved. The innovations rarely lead to innovations at higher organizational levels, from where they could disseminate further.

### A Forthcoming Paradigm Shift

**Figure 3:**
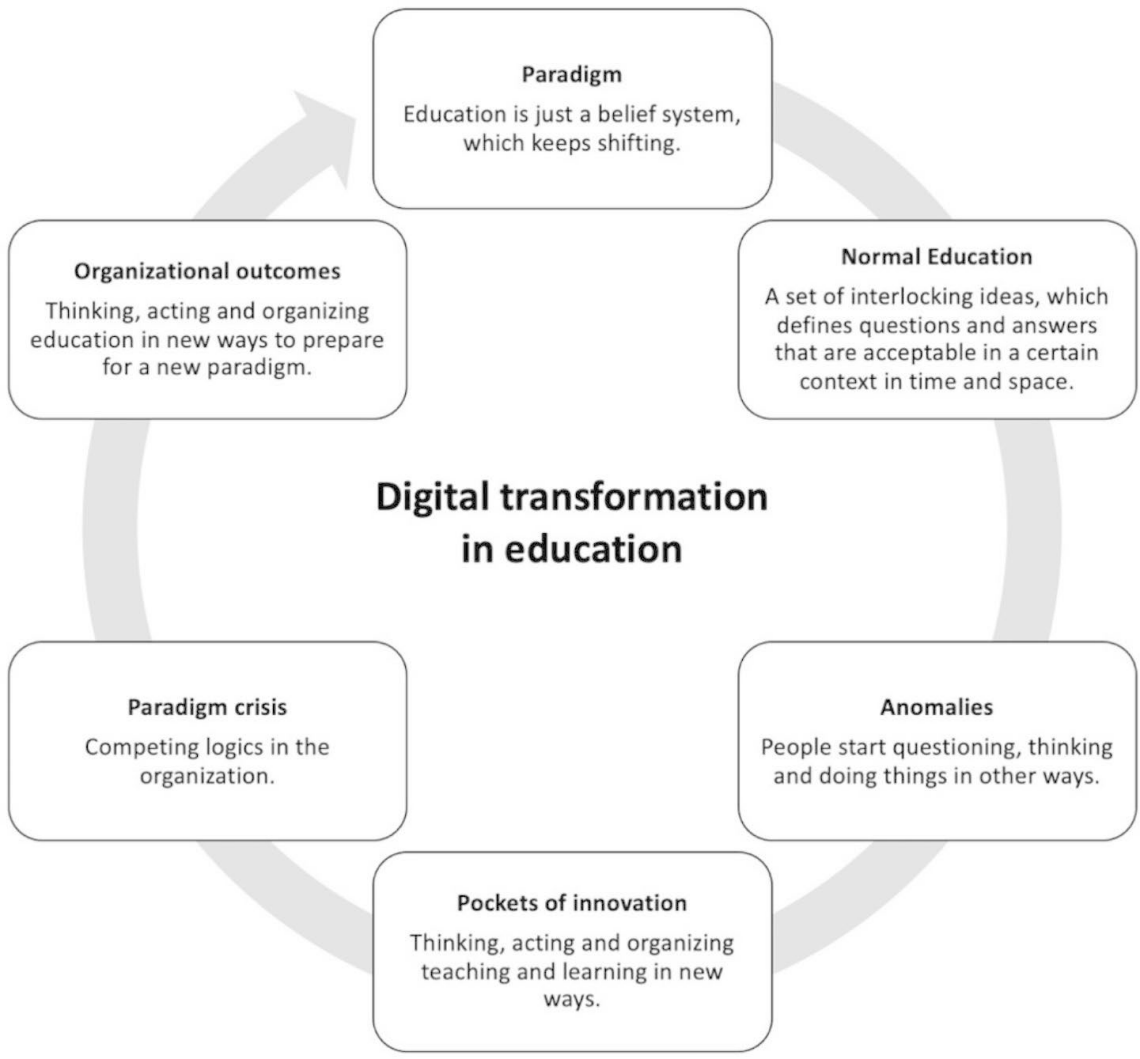
The process of a digital paradigm shifts in education

To further explain the subject position of entrepreneurs in the digital transformation of HE, we have made a model (Fig. 3) to support a more comprehensive system thinking. In this model, digitalization is still an anomaly that has not yet been socially accepted as normal education.

In contrast to Bygstad et al. (2022), who described forces that impede development, we found forces that *promote* digital transformation across institutional silos and barriers. *Loose couplings* made it possible for entrepreneurs in academic and technical positions to form networks and collaborate in teams. Here, they used their room for maneuver to find the necessary competences and skills to explore and innovate educational practices. Hence, they occupied subject positions in *pockets of innovation* that joined educational and administrative fields from other parts in their organization. When they sought to expand their room for maneuver, they often entered interpretative struggles and met resistance from faculties, leadership and management. That said, in our findings, MOOC entrepreneurs engaged in disruptive educational practices, which involved a potential for organizational change and agency. To illustrate the organizational process behind the development of new educational practices, we have outlined how paradigm shifts happen in the educational field of HE in the model below.

The model is based on our findings and inspired by Kuhn’s seminal research on paradigm shifts (Kuhn, 1970) and Macfarlane’s (2014) elaboration on *why* belief systems in universities have changed over the centuries. The model describes the shift in what we see as readiness, motivation and willingness to transform to MOOCs and online learning scenarios among groups of stakeholders: faculties managers leaders, and entrepreneurs.

According to Kuhn (op. cit.), science is not a linear progression of truth. Rather, science is just a belief system which keeps shifting. It comprises a set of interlocked ideas, which defines the questions and answers that are acceptable in a certain paradigm in time and space. What are considered acceptable questions and answers is described as *normal science* and paradigm shifts occur when faculty start thinking differently and act on these ideas. This is likely to happen in a situation where *anomalies* occur in numbers large enough to create competing logics in the organization. Anomalies are deviations from the norm that are difficult to maintain within the existing paradigm or within existing theories. Anomalies are usually phenomena that contradict existing scientific explanations. In other words, existing paradigms frame the way universities see the world, and universities are highly influenced by the space in time that they occupy.

We suggest *normal education* as a parallel term. Faculty build educational pathways for their students and teach their courses according to their own belief systems and experiences. Their experiences from years of schooling (K-12 and onwards) and working in academia have strongly contributed to forming this belief systems about education (Wiliam, 2011). In contrast, innovative entrepreneurial projects with successful and tangible outcomes disrupt normal education. Both in structure and numbers they constitute what Kuhn called an anomaly, a phenomenon that does not fit into contemporary practices, and hence prepare for a paradigm shift.

Macfarlane’s (2014) research on the historical development of universities from the 8th to the 21st century supplements Kuhn’s definition of a paradigm by trying to establish *why* belief systems keep shifting. According to Macfarlane, (1) seeing higher education in other ways is an academic activity that is very hard to pursue without academic independence, i.e., time to think, experts to discuss with and freedom to question anything. This is often the case in innovative entrepreneurial projects. Hence, the strength in independent universities, academic freedom and loose couplings. However, (2) new paradigms only occur when enough people start thinking and acting differently, in this case, when enough faculty start using new digital technologies in teaching and learning. In the next phase, the rest may follow, as was the case with Learning Management Systems (LMS) in the 1990s, and with online meetings during the Covid pandemic in the early 2020s. A main issue, according to Macfarlane, is also that (3) a paradigm shift is not a single, but a series of timely inventions that come together to create a new belief system. This took place when the Internet expanded (web 2.0 around 2004), users turned into active participants and lifelong learning for digital skills became in high demand. The belief system started to change (cf. paradigm crisis), as MOOC entrepreneurs explored the affordances of open platforms and experimented with digital learning designs.

In our findings, we see parallels with Kuhn’s theory on paradigms and Macfarlane’s historical research on why belief systems keep shifting. Macfarlane’s three conditions for change seem to align with our findings. Hence, we consider timely MOOC-entrepreneurial activities, as a driving force that has not yet reached a critical mass to prepare for a paradigm shift and extensive open online education in Norway. The urgency in preparing HEIs for flexible and scalable lifelong learning suggests, however, that a paradigm shift in HEI is on its way (see also Fullan 2021).

We are currently living the 4th Industrial revolution, which is triggered by technological solutions that have the potential to disrupt education in HEIs. Innovations that were initially funneled and spread from the United States due to a mix of investment capital, a series of technological inventions and the global nature of the Internet, provide a range of new synchronous and asynchronous on campus and online learning scenarios for students and faculties alike. Inventions like MOOC-platforms, digital tools for synchronous and asynchronous collaboration online, artificial intelligence (AI), virtual reality (VR), artificial reality (AR), chat-bots etc., are examples of new ways of thinking and doing, that emerge from questions embedded in technology industries and start-ups. These issues are quite different from pedagogical questions currently framing Education in HEIs in Norway. Our results suggest that MOOC entrepreneurship in pockets of innovation has the potential to expand, compete with logics in the organization and move teaching and learning towards a paradigm crisis. Consequently, MOOC entrepreneurship has the potential to change the belief system, residing in strategic, economic, and pedagogical questions pertaining to students’ learning and challenge normal education. Normal education is, however, upheld by interpretative struggles, discontinuity in projects and belief systems belonging to the exiting paradigm.

## Summary and Conclusion

In this study, we have explored how MOOC-entrepreneurs in HEIs can initiate and support transformative change in online teaching and learning. Three research questions served as steppingstones for our qualitative study of ten HEIs in Norway: (1) *What characterizes MOOC entrepreneurial activities in the current Norwegian sample? (2) What experiences do MOOC entrepreneurs draw from their involvement in educational change strategies? (3) How can transformative models describe digitalization processes in higher education?*

MOOC entrepreneurial activities were found in initiatives characterized by *change agency*, *hands-on experiences*, and *timely initiatives*. Change agency was rooted in areas of personal interest and converted into hands-on MOOC-production in a timely process influenced by external technological and pedagogical advances. MOOC entrepreneurial experiences were characterized by challenges related to both *feedback* and *funding* and to *institutional roles and positions* in in their organizations. MOOC-entrepreneurial activities were often based on what we have described as *single-loop entrepreneurship*, a series of projects without a long-term plan and with limited feedback from management and stakeholders, which narrowed the potential to contribute to organizational change. Their roles in transformational change and their location across institutional arrangements were to a large extent unresolved.

In this study we used thematic analysis to explore ten interviews with MOOC entrepreneurs in Norway. To sum up the most important results from this study, we point to certain organizational conditions of importance to digital agency, digital transformation and entrepreneurship (cf. Langseth et al., 2022).

Norwegian MOOC Entrepreneurs:

- have early adopter properties and capabilities.
- have a hands-on approach with limited research capacity.
- try out several educational MOOC formats.
- have know-how and skills to mobilize resources to work with MOOCs.
- use strategizing and networking skills and approaches to gain positions and resources.
- experience difficulties in resource mobilization at higher organizational levels.
- face boundary stagnation of MOOC practices and activities.

That said, we see the contours of a forthcoming paradigm shift in Norwegian higher education. In this disruptive process, we find that MOOC-entrepreneurs may facilitate a deeper understanding of how HEIs can adopt new technology to cater for a scalable and flexible education for all in the digital age. However, we also see competing logics and belief systems that impede this transformation.

## Recommendations

An important finding is that entrepreneurs miss access to a common national open platform. We recommend that such access be provided. This should happen at national levels, where government agencies take responsibility to finance and support such technological infrastructure. Another finding points to the problem that innovations are organized as one project at the time. This impedes continuity in organizational learning. Organizations must find new ways to evaluate project results to consider outcomes in the organization and avoid project overlap and subsequent burnout. Organizations should consider new roles to make sure that successful innovations are followed up and human capital taken care of in new reorganization processes. Research capacity should also be embedded in support systems to ensure a research-based process. Another result is the lack of a common language that transpires across institutional barriers. We found that different logics often lead to misunderstandings and unproductive communication. Here we also see the need for more research in organizational theory. Digitalization is a concept that has not yet found its form in institutional agency.

## Limitations

This is a qualitative study that is not statistically generalizable, and the data are limited to a Norwegian higher education context. Hence, we do not know how these phenomena would unfold in other countries with other institutional arrangements. A strength is, however, that it goes deep into the matter and finds new organizational patterns related to digital entrepreneurship. The study focuses on patterns and possibilities, where informants tell their story. One perspective that is not explored is the leadership’s take on these processes. These perspectives were, to a certain degree, intercepted from informants’ descriptions of their subject position and how they interacted with other stakeholders. Such a study remains to be done.

## Footnotes

1 SIKT, former UNIT and Bibsys, is a directorate that develops, manages and operates shared digital services and infrastructure, and contributes to secure digitization in the knowledge sector.
